# Medical treatment cost for Chinese inpatients with colorectal cancer by sites

**DOI:** 10.3389/fpubh.2025.1605887

**Published:** 2025-06-11

**Authors:** Zeng-Bao Hu, Jin-Ying Huang, Stuart McDonald, Bo-Xu Chen, Hao-Xun Mao, Zhou Wu, Xiao-Yu Dai, Hua Yu, Jian-Jiong Li, Yi Lin

**Affiliations:** ^1^Faculty of Humanities and Social Sciences, University of Nottingham Ningbo China, Ningbo, Zhejiang, China; ^2^College of International Economics and Trade, Ningbo University of Finance and Economics, Ningbo, Zhejiang, China; ^3^Department of Colorectal and Anal Surgery, Ningbo No. 2 Hospital, Ningbo, Zhejiang, China; ^4^Department of Clinical Nutrition, Ningbo No. 2 Hospital, Ningbo, Zhejiang, China

**Keywords:** China, colorectal cancer, medical costs, economic burden, stage at diagnosis

## Abstract

**Background:**

The increasing prevalence of colorectal cancer (CRC) is a challenge for China's healthcare system. Using hospitalization data from Ningbo, China, this study aims to estimate the medical treatment cost and cost structure of CRC based on tumor sites to gain insights with respect to the cost efficiency of early diagnosis.

**Methods:**

A retrospective observational study was performed in a real-life clinical setting of a tertiary hospital in Ningbo, China. Sociodemographic, clinicopathologic, and CRC medical treatment cost data were extracted from the inpatients' medical records. The study comprised inpatients aged above 18 diagnosed with CRC and received surgical treatment between 2020 and 2022. CRC costs were separated into six cost categories and analyzed separately by tumor site (rectum and colon). All cost data were measured by 2020 Chinese Yuan.

**Results:**

A total of 538 inpatients were included, where 63.9% were male, 67.5% were diagnosed with rectal cancer, and 47.2% were at Stages III and IV. Medical treatment costs of rectal cancer increased significantly from Stage I to Stage IV in all cost categories (*p* < 0.001), with percentage increases ranging from 70% to 120%, depending on cost category. Medication, materials, and examinations were the major sources of CRC costs for both rectal and colon cancers, with each accounting for 20%−30% of total costs, depending on tumor site and cancer stage.

**Conclusions:**

Targeted programs for the management and treatment of various tumor sites should be considered, as rectal cancer costs are more stage-sensitive than colon cancer. The large proportion of costs attributed to medication, materials, and examinations provides guidance to the government in regulating the healthcare market to alleviate the economic burden of CRC.

## 1 Introduction

Colorectal cancer (CRC) is the third most common cancer and the second leading cause of cancer-related death worldwide. It was estimated that there were 1.93 million new CRC cases and 0.94 million CRC deaths in 2020 (1). The global new cases and deaths are projected to increase to 2.2 million and 1.1 million by 2030, respectively (2). Europe and North America have higher incidence rates than most other parts of the world (1). In 2024, it was estimated that there were 0.15 million new cases in the United States and 0.15 million deaths in the European Union (3, 4). In China, the age-standardized incidence rate (ASIR) and mortality rate (ASMR) were 30.55 per 100,000 and 13.86 per 100, 000, respectively, in 2019 (5). Despite lower rates compared with developed countries such as the United States (ASIR: 41.86 per 100,000; ASMR: 14.77 per 100,000), China has the highest number of new CRC cases and CRC deaths worldwide due to its large population. In 2020, 0.55 million new CRC cases and 0.28 million CRC-related deaths occurred in China, accounting for 28.8% of newly diagnosed cases and 30.6% of CRC-related deaths worldwide, respectively (6, 7).

CRC has become a heavy economic burden in many countries. The estimated total medical costs of CRC were €19.1 billion in Europe in 2015 (8) and $24.3 billion in the United States in 2020 (9). In China, the economic burden of CRC reached 170.5 billion Chinese Yuan (CNY) in 2019, reaching 0.189% of the local GDP, and it is expected to reach 560 billion in 2030 without effective intervention (10).

CRC prevention programs and targeted treatment methods are therefore needed to alleviate the economic burden of CRC, which necessitates a thorough understanding of the structure of CRC medical treatment costs. Previous studies mostly focused on the medical costs of CRC in Western countries (11–14), while there have been limited studies focusing on a Chinese context. Existing studies on China have only estimated the total medical costs of CRC, rather than dissecting the costs into different categories, such as medication, examination, and surgery, to provide insights into the structure of costs (15, 16). By contrast, the aim of this study was to estimate the inpatient CRC medical treatment costs by cost categories (i.e., material cost, medication cost, surgery cost, treatment cost, examination cost, and hospital service cost), and compare the costs by patient sociodemographic characteristics, tumor sites, and cancer stages, in a real-life clinical setting of a tertiary hospital in Ningbo, Zhejiang, China. The CRC prevalence in Zhejiang has caused a heavier economic burden than other Chinese provinces (17, 18), making it a good example to study the costs of CRC treatment and relevant contributors.

## 2 Methodology and data

### 2.1 Study design and patients

This study provides a retrospective observational study using the pooled data from a tertiary hospital located in Ningbo, Zhejiang, China. This study was approved by the Ethics Committee of the hospital (No. YJ-NBEY-KY-2023-060-01). Patients who satisfied the following inclusion criteria were eligible for the study: (1) primary diagnosis, hospital admission, and surgical treatment for CRC occurred between January 1 2020 and December 31 2022; (2) age of subjects was above or equal to 18 years of age at the time of inclusion in the data set; (3) information on cancer stage and CRC-related costs during hospitalization was available; and (4) other individual clinical information including age, gender, tumor site, and usage of traditional Chinese medicine (TCM), were available. According to the TNM staging system of the Union for International Cancer Control (UICC) (19), patients were divided into four cancer stages (I, II, III, and IV). Based on the age at diagnosis, patients were divided into three age groups: < 60, 60–79, and >79. Regarding whether patients took TCM during hospitalization, subjects were classified into two groups: those without TCM usage and those with TCM usage.

### 2.2 Cancer treatment costs

The total treatment costs in this study referred to the total medical costs incurred by CRC patients during hospitalization. Total treatment costs were then separated into six categories: material costs, medication costs, surgery costs, treatment costs, examination costs, and hospital service costs. Material costs included the expenses on medical instruments and equipment used during hospitalization and surgeries (e.g., syringes, medical tubing, medical needles, surgery instruments, etc.). Medication costs included all the drugs used during hospitalization, including prescription drugs and over-the-counter (OTC) drugs. Medication costs did not include the expenses incurred from patients using traditional Chinese medicine (TCM). Surgery costs included the costs of the operation and anaesthetization. Treatment costs included the expense of providing inpatients with necessary care during hospitalization (e.g., injection, intubation, dressing change, etc.). Examination costs included the costs of all medical examinations, including radiology, nuclear medicine, endoscopy, and laboratory testing, incurred by patients during hospitalization. Hospital service costs included the costs of nursing service, hospital accommodation, blood transfusion, consultation, and TCM. All costs were measured in CNY based on the Consumer Price Index (CPI) of China using 2020 as the base year.

### 2.3 Statistical analysis

The pooled sample data were analyzed in the study. CRC-related medical costs and sociodemographic and clinicopathologic characteristics of inpatients were described and analyzed in this study. CRC-related costs were presented as means with 95% confidential intervals (CIs). Gender, age group, TCM usage, and cancer stage were reported as numbers with percentages. The Shapiro-Wilk test was used to test for the normality of continuous variables, and the non-parametric Jonckheere-Terpstra test was used to compare their statistical differences between groups, given that the tested samples were non-normally distributed. The Chi Square test was used to compare the statistical differences in categorical variables across groups. Separate analyses were undertaken for colon and rectal cancers due to their biological and clinical differences (20). All statistical tests were two-tailed, and *p* < 0.05 was considered statistically significant. Statistical analyses were performed using STATA 17.0 (2021).

## 3 Results

**Figure 1:**
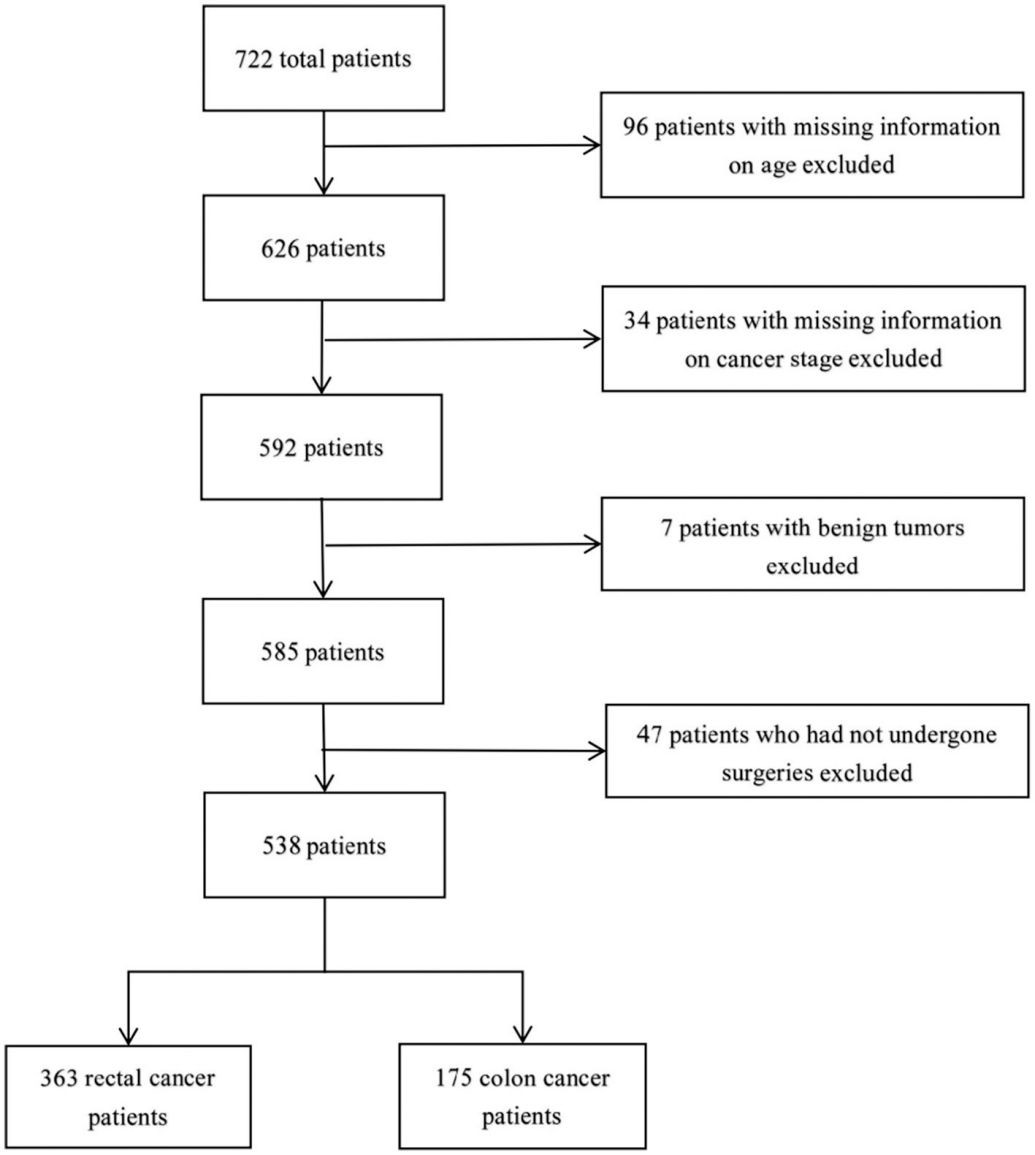
Flow chart of inclusion of the study sample.

The data contained a total of 722 inpatients diagnosed with CRC between 2020 and 2022, with 538 subjects satisfying the criteria for inclusion in this study (Figure 1). A total of 538 inpatients were included in this study, with 63.9% were male, 53.7% aged between 60 and 79, 83.6% elected not to take TCM during hospitalization, and 67.5% diagnosed with rectal cancer (Table 1). The proportions of patients at Stages I, II, III, and IV were 21.2, 31.6, 35.5, and 11.7%, respectively. Patients with rectal cancer had a significantly younger age structure (*p* < 0.001), with 22.9% under the age of 59, compared to 10.8% with colon cancer. There were significant differences in cancer stage between colon cancer and rectal cancer (*p* < 0.001), as higher percentages of Stage III (36.4%) and Stage II (37.1%) were found in rectum cancer and colon cancer, respectively. Figure 1 details the sample procedure and inclusion process.

**Table 1:** Colorectal cancer patient characteristic and medical expenditures by tumor site.

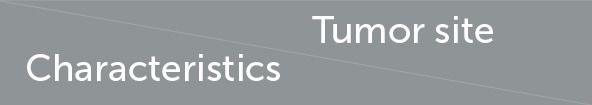	**Total sample**	**Rectum**	**Colon**	**P^a^**
N (%)				
**Patient**				
Number of patients	538 (100.0%)	363 (67.5%)	175 (32.5%)	
**Gender**				0.984
Male	344 (63.9%)	232 (63.9%)	112 (64.0%)	
Female	194 (36.1%)	131 (36.1%)	63 (36.0%)	
**Age group**				< 0.001
≤ 59	102 (19.0%)	83 (22.9%)	19 (10.9%)	
60-79	289 (53.7%)	197 (54.2%)	92 (52.6%)	
≥80	147 (27.3%)	83 (22.9%)	64 (36.6%)	
**Traditional Chinese medicine usage**				0.010
No	450 (83.6%)	314 (86.5%)	136 (77.7%)	
Yes	88 (16.4%)	49 (13.5%)	39 (22.3%)	
**Cancer stage**				0.001
TNM I	114 (21.2%)	92 (25.3%)	22 (12.6%)	
TNM II	170 (31.6%)	105 (28.9%)	65 (37.1%)	
TNM III	191 (35.5%)	132 (36.4%)	59 (33.7%)	
TNM IV	63 (11.7%)	34 (9.4%)	29 (16.6%)	
Mean [95% CI]				
Total cost	37946.13	32515.61	49210.59	< 0.001
	[36006.15-39886.11]	[30240.10-34791.11]	[46156.31-52264.86]	
Material cost	9101.29	7298.04	12841.73	< 0.001
	[8350.62-9851.95]	[6588.29-8007.79]	[11185.48-14497.98]	
Medication cost	9830.81	8559.71	12467.45	< 0.001
	[9215.52-10446.11]	[7821.08-9298.34]	[11455.59-13479.31]	
Surgery cost	5091.29	4726.53	5847.92	< 0.001
	[4914.98-5267.60]	[4506.51-4946.55]	[5586.55-6109.28]	
Treatment cost	3070.79	2599.87	4047.62	< 0.001
	[2843.30-3298.29]	[2316.76-2882.98]	[3707.55-4387.69]	
Examination cost	8421.13	7073.36	10663.42	< 0.001
	[7845.50-8636.76]	[6607.33-7539.39]	[10063.10-11263.74]	
Hospital service cost	2521.28	2177.91	3233.53	< 0.001
	[2373.43-2669.14]	[1995.83-2360.00]	[3013.48-3453.58]	

Values are expressed as frequency (%), or as mean [95% CI].

Costs are measured by 2020 CNY (¥).

^a^Comparisons are made between the rectum group and the colon group, using the Chi-2 Test for categorical variables and the Jonckheere–Terpstra Test for continuous variables, respectively.

Results are presented as *P*-values.

The mean total medical cost for CRC treatment was ¥37946.13 per patient, with ¥9101.29 for materials, ¥9830.81 for medications, ¥5091.29 for surgeries, ¥3070.79 for treatment, ¥8421.13 for examinations, and ¥2521.28 for hospital services (Table 1). The mean costs across all cost categories were significantly higher for colon cancer than rectal cancer (*p* < 0.001).

Table 2 displays the changes in the cost structures of CRC medical costs from 2020 to 2022. The proportions of material cost and surgery cost increased, while the proportion of medication costs decreased. The proportions of other cost categories were relatively stable, with minor changes.

**Table 2:** Cost structure of medical expenditures for colorectal cancer from 2020 to 2022.

**Category**	**Year**			**Annual growth rate**
	**2020**	**2021**	**2022**	
Material cost	20.87%	23.49%	26.92%	13.58%
Medication cost	29.69%	26.13%	23.06%	−11.87%
Surgery cost	11.99%	13.47%	14.52%	10.07%
Treatment cost	9.01%	7.57%	7.97%	−5.35%
Examination cost	21.27%	23.03%	20.91%	−0.47%
Hospital service cost	7.18%	6.32%	6.61%	−3.69%

Table 3 summarizes the mean cost by patient characteristics for inpatients with rectal and colon cancers. The total cost for rectal cancer was found to be significantly higher in male patients (*p* = 0.012) and older patients (*p* < 0.001), but not for colon cancer. The total cost of patients was significantly higher for those using TCM for both rectal (*p* = 0.002) and colon (*p* = 0.005) cancers. The medical treatment cost increased significantly with stage levels for rectal cancer in any cost category (*p* < 0.001). By contrast, no significant difference in the medical treatment cost across cancer stages was observed for colon cancer.

**Table 3:** Mean cost of rectal cancer and colon cancer by cost category and demographic variables.

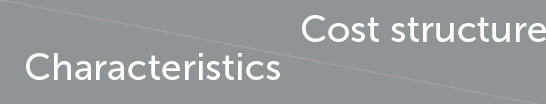	**Total cost**	**Material cost**	**Medication cost**	**Surgery cost**	**Treatment cost**	**Examination cost**	**Hospital Service cost**
**Rectal cancer**							
**Total sample**	32515.61	7298.04	8559.71	4726.53	2599.87	7073.36	2177.91
**Gender**							
Male	34180.03	7832.27	9111.27	4766.18	2740.17	7372.91	2263.52
Female	29567.91	6351.92	7582.89	4656.30	2351.41	6542.87	2026.30
*P*-value	0.012	0.023	0.004	0.576	0.068	0.046	0.018
**Age group**							
≤ 59	25765.11	5298.24	7355.39	4081.35	1851.07	5294.00	1775.20
60-79	29226.80	6184.01	7946.34	4322.52	2378.13	6319.25	2012.48
≥80	47072.05	11942.00	11219.86	6330.62	3874.97	10642.60	2973.27
*P*-value	< 0.001	< 0.001	< 0.001	< 0.001	< 0.001	< 0.001	< 0.001
**TCM**							
Yes	41450.65	9601.37	10690.49	5261.27	3741.00	9239.40	2777.01
No	31121.28	6938.60	8227.20	4643.08	2421.80	6735.35	2084.42
*P*-value	0.002	0.010	0.018	0.026	0.004	< 0.001	< 0.001
**Cancer stage**							
TNM I	25622.49	5439.52	6642.04	4074.17	1920.94	5686.55	1750.98
TNM II	30789.59	6620.54	8186.59	4588.53	2456.52	6638.79	2216.57
TNM III	34076.92	7908.84	8602.41	5069.41	2672.65	7605.61	2148.45
TNM IV	50436.33	12047.91	14735.22	5586.72	4597.14	10101.58	3328.13
*P*-value	< 0.001	< 0.001	< 0.001	< 0.001	< 0.001	< 0.001	< 0.001
**Colon cancer**							
Total sample	49210.59	12841.73	12467.45	5847.92	4047.62	10663.42	3233.53
**Gender**							
Male	49066.41	13052.68	12551.10	5921.36	3888.86	10468.84	3080.45
Female	49466.89	12466.70	12318.74	5717.40	4329.86	11009.33	3505.67
*P*-value	0.702	0.326	0.621	0.349	0.334	0.518	0.455
**Age group**							
≤ 59	48649.63	18028.95	10496.13	5522.23	3220.13	8533.93	2791.30
60-79	50582.05	13239.09	13346.92	6125.04	4039.11	10391.45	3349.92
≥80	47405.64	10730.57	11788.45	5546.24	4305.52	11686.56	3197.50
*P*-value	0.405	0.586	0.415	0.083	< 0.001	< 0.001	0.159
**TCM**							
Yes	55237.53	15073.77	13531.95	6134.84	4483.04	12276.29	3605.64
No	47482.27	12201.66	12162.19	5765.64	3922.76	10200.90	3126.82
*P*-value	0.005	0.096	0.043	0.114	0.082	< 0.001	0.031
**Cancer stage**							
TNM I	46557.84	12457.94	10809.08	6374.00	3533.31	9875.55	3262.98
TNM II	48095.37	11717.19	12629.61	5697.59	4054.04	10695.51	3229.49
TNM III	52224.37	15426.79	12676.82	5871.22	4116.04	10841.64	3210.47
TNM IV	47591.14	10394.16	12936.08	5738.37	4284.22	10826.59	3267.15
*P*-value	0.505	0.087	0.743	0.358	0.617	0.995	0.413

Costs are measured by 2020 CNY (¥).

Comparisons between groups are conducted using the Jonckheere–Terpstra Test. Results are presented as *P*-values.

**Figure 2:**
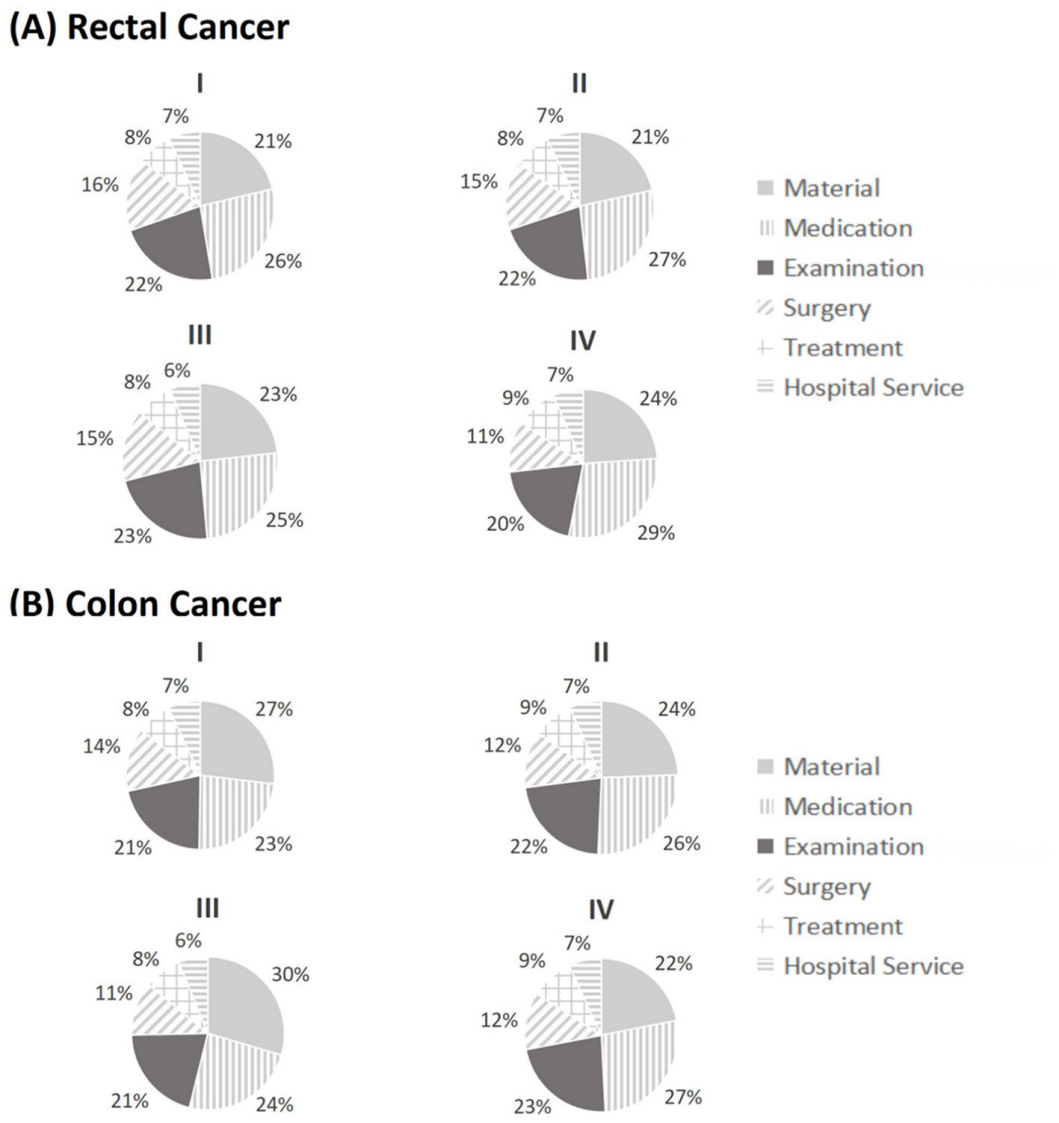
Cost structure of medical expenditures for rectal and colon cancer by stage classification (I, II, III, IV). Medical expenditures were separated into six categories including material cost, medication cost, examination cost, surgery cost, treatment cost, and hospital service cost.

Figure 2 shows the cost structures of medical treatment costs by cancer stage and tumor site. Medical costs for rectal and colon cancer exhibited similar cost structures, with medication, examination, and materials as the three major cost drivers, contributing to over 70% of total costs. Medication was the largest cost source for rectal cancer, ranging from 25% to 29%, depending on the cancer stage, and it was also the largest source for colon cancer at Stages II (26%) and IV (27%). However, the largest cost source for colon cancer at Stages I (27%) and III (30%) was material cost. The proportion of examination cost was relatively stable for both rectal and colon cancers, ranging from 20% to 23%, depending on the cancer stage and tumor site. Treatment and hospital services remained the least important contributors for both rectal and colon cancers, with stable proportions of 7%−9%. When comparing the cost structures between rectal and colon cancers, the proportion of material cost was substantially higher in colon cancer than in rectal cancer at Stages I, II, and III, except for Stage IV. The differences in other costs were relatively small.

## 4 Discussion

This study found that the mean costs were significantly higher in colon cancer across all cost categories than in rectal cancer. This contrasts with previous studies, some of which reported that the costs for rectal cancer were higher than those for colon cancer (15, 21, 22), while others found no significant difference between the costs of rectal and colon cancers (12, 23). Two reasons may account for this difference. First, rectal cancer is more likely to be diagnosed at an earlier stage, due to specific initial symptoms such as rectal bleeding (24). As a result, the proportion of patients at Stage I was higher in the rectal cancer sample than the colon cancer sample (25.3% vs. 12.6%) in this study, leading to lower medical costs. In contrast, the proportions of patients at an early stage were similar between rectal and colon cancers in previous literature, such as 59% for rectal cancer vs. 58% for colon cancer (22) or 49% for rectal cancer vs. 51% for colon cancer (12). Second, older patients are more likely to experience complications and comorbidities (25, 26), and in our study, the proportion of patients aged above 80 was higher for colon cancer than for rectal cancer (36.6% vs. 22.9%), which may have raised medical costs.

This study also found that the mean cost of CRC increased with the TNM stage of diagnosis, which is consistent with previous studies (23, 27, 28). However, colon and rectal cancers showed different cost features along with cancer stages. For the patients with rectal cancer, costs in all categories increased significantly from Stage I to Stage IV, as later stages usually require more complicated treatment strategies and additional medication to combat complications and comorbidities. Moreover, rectal cancer at Stages III and IV usually involves resection of a larger part of the rectum and adjacent tissues or organs invaded by cancer cells (29), which could cause greater damage to the digestive and immune systems and therefore raise relevant costs. For colon cancer, medical costs generally increased from Stage I to Stage IV in our study, although the differences between costs across stages were not significant. It may be because there were fewer colon cancer patients than rectal cancer patients included in our study (175 vs. 363), and the limited number of observations may influence the power of the estimation.

Medication, materials, and examinations were the three major sources of medical costs for CRC, which was similar to a recent research based on a tertiary hospital in Shanghai (30). The proportions of medication cost in total costs varied from 23% to 29%, depending on the cancer stage and tumor site. This was similar to some foreigner studies conducted in Serbia (31%) (13) and the EU (27%) (11), but differed from previous studies in France (5%) (14), Finland (12% in primary disease state to 60% in metastatic state) (12), Jordan (54%) (23), and Vietnam (62%) (31). Such disparities may be attributed to the differences in drug prices and pharmacy prescription regulations across countries. The proportion of examination cost in our study ranged from 20% to 23%, which was comparable to studies in Serbia (18%) (13) and Jordan (22%) (23). The surgery cost made up 11% to 16% of total costs in our study, and the proportions were comparable to those in Serbia (11%−13%) (13), and Vietnam (12%) (31), but differed from studies in Jordan (5%) (23) and the US (53%) (32). The proportion of medication costs in our study witnessed a dramatically decrease from 2020 to 2022, with an annual rate of −11.87%. It is worth noting that the Chinese government has been exerting efforts to regulate the medical market to reduce the economic burden of cancer drugs for its citizens, which could partly explain the decreasing proportion of medication costs in our study. However, the proportion of surgery costs increased by 10.07% annually from 2020 to 2022, and this might be attributed to the increased material costs (20.87% in 2020 to 26.92% in 2022).

CRC-related costs were found to be related to patient characteristics. Male patients had significantly higher medical costs than females for rectal cancer, but not for colon cancer. This might be due to the narrower and deeper pelvises of males, which make the treatment of rectal cancer more difficult and therefore costlier (33). In addition, rectal cancer was found to be more sensitive to smoking (34) and alcohol consumption (35) than colon cancer. Males were found more likely than females to have unhealthy lifestyle habits such as alcohol consumption and smoking (36, 37) that increase the risk of rectal cancer, which may result in higher medical costs. Patients over 80 showed significantly higher costs for rectal cancer in all cost categories than patients below 60, probably due to weaker physical function and poorer immune systems. This feature was also observed in the treatment cost and examination cost for colon cancer. However, previous studies in Western countries have reported a contrasting feature, as seen in France (14), Finland (12), and the USA (38). This highlights the complex relationship between age and CRC-related costs, which is worth further investigation as a research topic. Rectal cancer patients who used TCM had significantly higher CRC-related costs than those who did not. This is probably because patients with TCM usage had a higher average age (74.51 vs. 69.38), and TCM was more likely to be used as a complementary and alternative therapy for older patients (39).

The findings of this study may have important implications for China's health policies. The stage at diagnosis was found to play a critical role in influencing the costs of rectal and colon cancers. CRC patients at later stages (III and IV) had higher medical costs than patients at earlier stages (I and II). Thus, CRC prevention measures like screening should be encouraged to be implemented for early diagnosis. CRC screening has been widely implemented as a basic public health welfare in many developed countries (40). In comparison, the current overall CRC screening uptake in China is still limited because of its huge population and limited healthcare resources (7). The limited participation in CRC screening of Chinese citizens had led to later diagnosis and thus increased the economic burden. In 2019, the Healthy China Action by the Chinese government was proposed to increase the cancer screening coverage of the overall population (16). To achieve this, the Chinese government may develop risk-prediction models to identify the population at high risk of CRC and introduce health awareness campaigns to encourage participation in CRC screening programs, particularly among population with higher CRC risks (41). With respect to the cost structure of CRC, medication, materials, and examinations are the three major sources of medical expenditures for rectal cancer and colon cancer. The government may incorporate more types of medication and medical materials into the centralized purchasing system to decrease the economic burden for CRC patients caused by materials and medication. Improvements may also be made in surgical and treatment strategies and technologies to reduce the economic burden of CRC treatment. Additionally, this study also showed the variations in CRC-related costs by patient age and gender, suggesting the potential improvements that could be made in patient treatment strategies for heterogeneous patients.

This study has some strengths relative to previous studies examining the costs of treating CRCs. First, it explored the cost structure of CRC-related costs by dividing costs into six sub-categories, providing more detailed and specific insights into CRC costs. Second, the sample was separated by tumor site (rectum and colon) to better reflect the heterogeneity effect on CRC costs by tumor site. By contrast, previous studies grouped colon cancer and rectal cancer together, without considering their differences in anatomy and treatment (15, 16, 30). However, some limitations should be acknowledged. First, due to data limitations, this study did not include outpatient costs such as radiotherapy and chemotherapy. Second, we only included patients diagnosed with CRC and received treatment in the study hospital, while patients who received multiple treatments and those who were transferred to other hospitals were not considered. Third, we did not take into account the impacts of patients' social-economic status (SES) on CRC medical costs. Fourth, this study was based on Ningbo in Eastern China, while the incidence rate and CRC medical costs may vary due to regional differences in lifestyle, environment, and SES across different parts of China. This may affect the representativeness and generalizability of our results. A well-designed research with a larger population that includes both direct and indirect costs should be conducted in the future to construct an estimate and prediction of the cost burden on the health system.

## 5 Conclusions

This retrospective study estimated the costs of CRC patients by cost category in real-life clinical settings in Ningbo, China. Medication, materials, and examinations were the main cost drivers of CRC treatment. The cost was significantly higher for colon cancer, which was typically identified at a later stage. The mean medical cost increased significantly from Stage I to Stage IV for rectal cancer. The findings suggest implications for the management of rectal and colon cancers, specifically the importance of early screening among the higher risk population for colon cancer and tailored treatment strategies for different tumor sites. Future studies should include the outpatient and indirect costs of CRC, and data from other regions of China.

## Data Availability

The data is not publicly available due to privacy or ethical restrictions. If there is a reasonable request, it can be obtained from the corresponding authors.

## References

[1] Sung H Ferlay J Siegel RL Laversanne M Soerjomataram I Jemal A. Global cancer statistics 2020: GLOBOCAN estimates of incidence and mortality worldwide for 36 cancers in 185 countries. CA Cancer J Clin. (2021) 71:209–49. 10.3322/caac.21660 33538338

[2] Jafari A Hosseini FA Jalali FS. A systematic review of the economic burden of colorectal cancer. Health Sci Rep. (2024) 7:e70002. 10.1002/hsr2.70002 39170890 PMC11336656

[3] Santucci C Mignozzi S Malvezzi M Boffetta P Collatuzzo G Levi F. European cancer mortality predictions for the year 2024 with focus on colorectal cancer. Ann Oncol. (2024) 35:308–16. 10.1016/j.annonc.2023.12.003 38286716

[4] Siegel RL Giaquinto AN Jemal A. Cancer statistics, 2024. CA Cancer J Clin. (2024) 74:12–49. 10.3322/caac.21820 38230766

[5] Li Q Wu H Cao M Li H He S Yang F. Colorectal cancer burden, trends and risk factors in China: a review and comparison with the United States. Chin J Cancer Res. (2022) 34:483. 10.21147/j.issn.1000-9604.2022.05.08 36398126 PMC9646460

[6] Chen H Lu B Dai M. Colorectal cancer screening in China: status, challenges, and prospects-China, 2022. China CDC Weekly. (2022) 4:322–8. 10.46234/ccdcw2022.077 35548454 PMC9081894

[7] Li N Lu B Luo C Cai J Lu M Zhang Y. Incidence, mortality, survival, risk factor and screening of colorectal cancer: a comparison among China, Europe, and northern America. Cancer Lett. (2021) 522:255–68. 10.1016/j.canlet.2021.09.034 34563640

[8] Henderson RH French D Maughan T Adams R Allemani C Minicozzi P. The economic burden of colorectal cancer across Europe: a population-based cost-of-illness study. Lancet Gastroenterol. Hepatol. (2021) 6:709–22. 10.1016/S2468-1253(21)00147-3 34329626

[9] US CDC (US Centers for Disease Control and Prevention). Health and Economic Benefits of Colorectal Cancer Interventions. (2020). Available online at: https://www.cdc.gov/chronicdisease/programs-impact/pop/colorectal-cancer.htm (accessed November 19, 2023).

[10] Wang H Li YJ Lei L Liu CC Chen WQ Dai M. Estimating the economic burden of colorectal cancer in China, 2019–2030: a population-level prevalence-based analysis. Cancer Med. (2024) 13:e6787. 10.1002/cam4.6787 38112048 PMC10807552

[11] Luengo-Fernandez R Leal J Gray A Sullivan R. Economic burden of cancer across the European Union: a population-based cost analysis. Lancet Oncol. (2013) 14:1165–74. 10.1016/S1470-2045(13)70442-X 24131614

[12] Färkkilä N Torvinen S Sintonen H Saarto T Järvinen H Hänninen J. Costs of colorectal cancer in different states of the disease. Acta Oncol. (2015) 54:454–62. 10.3109/0284186X.2014.985797 25519708

[13] Vekic B Dragojevic-Simic V Jakovljevic M Pilipovic F Simic R Zivic R. Medical cost of colorectal cancer services in Serbia between 2014 and 2017: national data report. Front. Pharmacol. (2019) 10:526. 10.3389/fphar.2019.00526 31156439 PMC6530405

[14] Bouvier V Reaud JM Gignoux M Launoy G. Cost of diagnostic and therapeutic management of colorectal cancer according to stage at diagnosis in the Calvados Département, France. Eur J Health Econ. (2003) 4:102–6. 10.1007/s10198-002-0160-3 15609176

[15] Wong CK Lam CL Poon JT McGhee SM Law WL Kwong DL. Direct medical costs of care for Chinese patients with colorectal neoplasia: a health care service provider perspective. J Eval Clin Pract. (2012) 18:1203–10. 10.1111/j.1365-2753.2011.01776.x 22111837

[16] Shi JF Wang L Ran JC Wang H Liu CC Zhang HZ. Clinical characteristics, medical service utilization, and expenditure for colorectal cancer in China, 2005 to 2014: overall design and results from a multicenter retrospective epidemiologic survey. Cancer. (2021) 127:1880–93. 10.1002/cncr.33445 33784413

[17] Cai Y Xue M Chen W Hu M Miao Z Lan L. Expenditure of hospital care on cancer in China, from 2011 to 2015. Chin J Cancer Res. (2017) 29:253. 10.21147/j.issn.1000-9604.2017.03.11 28729776 PMC5497212

[18] Zhang L Cao F Zhang G Shi L Chen S Zhang Z. Trends in and predictions of colorectal cancer incidence and mortality in China from 1990 to 2025. Front Oncol. (2019) 9:98. 10.3389/fonc.2019.00098 30847304 PMC6393365

[19] Brierley JD Gospodarowicz MK Wittekind C eds. TNM Classification of Malignant Tumours. Hoboken, NJ: John Wiley & Sons (2017).

[20] Tamas K Walenkamp AME de Vries EGE van Vugt MATM Beets-Tan RG van Etten B. Rectal and colon cancer: not just a different anatomic site. Cancer Treat Rev. (2015) 41:671–9. 10.1016/j.ctrv.2015.06.007 26145760

[21] Jansman FG Postma MJ Brouwers JR. Cost considerations in the treatment of colorectal cancer. Pharmacoeconomics. (2007) 25:537–62. 10.2165/00019053-200725070-00002 17610336

[22] Haug U Engel S Verheyen F Linder R. Estimating colorectal cancer treatment costs: a pragmatic approach exemplified by health insurance data from Germany. PLoS ONE. (2014) 9:e88407. 10.1371/journal.pone.0088407 24586324 PMC3929363

[23] Alefan Q Malhees R Mhaidat N. Direct medical cost associated with colorectal cancer in north of Jordan. Curr Probl Cancer. (2017) 41:371–81. 10.1016/j.currproblcancer.2017.05.001 28629637

[24] Korsgaard M Pedersen L Sørensen HT Laurberg S. Reported symptoms, diagnostic delay and stage of colorectal cancer: a population-based study in Denmark. Colorectal Dis. (2006) 8:688–95. 10.1111/j.1463-1318.2006.01014.x 16970580

[25] Weerink LB Gant CM van Leeuwen BL de Bock GH Kouwenhoven EA Faneyte IF. Long-term survival in octogenarians after surgical treatment for colorectal cancer: prevention of postoperative complications is key. Ann Surg Oncol. (2018) 25:3874–82. 10.1245/s10434-018-6766-1 30244418 PMC6245105

[26] Gheybi K Buckley E Vitry A Roder D. Occurrence of comorbidity with colorectal cancer and variations by age and stage at diagnosis. Cancer Epidemiol. (2022) 80:102246. 10.1016/j.canep.2022.102246 36067574

[27] Francisc S Guzzinati S Mezzetti M Crocetti E Giusti F Miccinesi G. Cost profiles of colorectal cancer patients in Italy based on individual patterns of care. BMC Cancer. (2013) 13:1–11. 10.1186/1471-2407-13-329 23826976 PMC3706387

[28] Gitlin M Fadli E Chung KC. Increased healthcare costs by later stage cancer diagnosis. BMC Health Serv Res. (2022) 22:1–12. 10.1186/s12913-022-08457-6 36096813 PMC9469540

[29] National Health Commission of PRC, Society Society of Oncology, Chinese Medical Association. National health commission guidelines for diagnosis and treatment of colorectal cancer 2023 in China (English version). Chin J Cancer Res. (2023) 35:197–232. 10.21147/j.issn.1000-9604.2023.03.01 37440823 PMC10334494

[30] Lin Y Lv J Zhou Y Liu Y. Analysis on the structural variation and influencing factors of hospitalization expenses of patients with colorectal cancer surgery. Chin Med Record. (2024) 25:72–6.

[31] Le NQ Vo TQ Doan TD (2019). Analyzing the variation in treatment costs for colorectal cancer (CRC): a retrospective study to assess an underlying threat among the Vietnamese. J Pak Med Assoc. (2019) 69:S34-40. 31369532

[32] Warren JL Yabroff KR Meekins A Topor M Lamont EB Brown ML. Evaluation of trends in the cost of initial cancer treatment. J Natl Cancer Inst. (2008) 100:888–97. 10.1093/jnci/djn175 18544740 PMC3298963

[33] Kim JS Cho SY Min BS Kim NK. Risk factors for anastomotic leakage after laparoscopic intracorporeal colorectal anastomosis with a double stapling technique. J Am Coll Surg. (2009) 209:694–701. 10.1016/j.jamcollsurg.2009.09.021 19959036

[34] Botteri E Iodice S Bagnardi V Raimondi S Lowenfels AB Maisonneuve P. Smoking and colorectal cancer: a meta-analysis. JAMA. (2008) 300:2765–78. 10.1001/jama.2008.839 19088354

[35] Pedersen A Johansen C Grønbaek M. Relations between amount and type of alcohol and colon and rectal cancer in a Danish population based cohort study. Gut. (2003) 52:861–7. 10.1136/gut.52.6.861 12740343 PMC1773681

[36] Wang S Ungvari GS Forester BP Chiu HFK Wu Y Kou C. Gender differences in general mental health, smoking, drinking and chronic diseases in older adults in Jilin province, China. Psychiatry Res. (2017) 251:58–62. 10.1016/j.psychres.2017.02.007 28189080

[37] Kim JH Lee S Chow J Lau J Tsang A Choi J. Prevalence and the factors associated with binge drinking, alcohol abuse, and alcohol dependence: a population-based study of Chinese adults in Hong Kong. Alcohol Alcohol. (2008) 43:360–70. 10.1093/alcalc/agm181 18230698

[38] Tramontano AC Chen Y Watson TR Eckel A Hur C Kong CY. Racial/ethnic disparities in colorectal cancer treatment utilization and phase-specific costs, 2000–2014. PLoS ONE. (2020) 15:e0231599. 10.1371/journal.pone.0231599 32287320 PMC7156060

[39] Sun J Wei Y Wang J Hou M Su L. Treatment of colorectal cancer by traditional Chinese medicine: prevention and treatment mechanisms. Front Pharmacol. (2024) 15:1377592. 10.3389/fphar.2024.1377592 38783955 PMC11112518

[40] Schreuders EH Ruco A Rabeneck L Schoen RE Sung JJ Young GP. Colorectal cancer screening: a global overview of existing programmes. Gut. (2015) 64:1637–49. 10.1136/gutjnl-2014-309086 26041752

[41] Xu LL Lin Y Han LY Wang Y Li JJ Dai XY. Development and validation of a prediction model for early screening of people at high risk for colorectal cancer. World J Gastroenterol. (2024) 30:450. 10.3748/wjg.v30.i5.450 38414586 PMC10895599

