# Supplementary material for: Medical treatment cost for Chinese inpatients with colorectal cancer by sites
Source: Front Public Health. 2025 Jun 11;13:1605887. doi: 10.3389/fpubh.2025.1605887 (PMC12187665; doi:10.3389/fpubh.2025.1605887)
Supplement: Supplementary file 1 [file Table_1.docx]

Supplementary Material

**Supplementary Table 1.** Colorectal Cancer Medical Expenditures by Tumor Site Measured in EUR and USD

|  | **Total** | **Rectum** | **Colon** | **P**^a^ |
| --- | --- | --- | --- | --- |
| Mean [95% CI]^b^ | | | | |
| **Total Cost** | 5073.73 | 4354.65 | 6565.31 | <0.001 |
|  | [4814.24-5333.22] | [4051.04-4658.25] | [6152.47-6978.14] |  |
| **Material Cost** | 1222.34 | 982.02 | 1720.83 | <0.001 |
|  | [1120.77-1323.90] | [886.23-1077.81] | [1495.75-1945.91] |  |
| **Medication Cost** | 1308.78 | 1141.10 | 1656.60 | <0.001 |
|  | [1228.07-1389.49] | [1044.26-1237.94] | [1523.91-1789.29] |  |
| **Surgery Cost** | 682.88 | 635.18 | 781.81 | <0.001 |
|  | [658.65-707.11] | [605.00-665.37] | [745.26-818.36] |  |
| **Treatment Cost** | 410.02 | 347.60 | 539.48 | <0.001 |
|  | [379.69-440.34] | [309.97-385.24] | [493.71-585.24] |  |
| **Examination Cost** | 1100.91 | 946.55 | 1421.09 | <0.001 |
|  | [1048.14-1153.68] | [884.34-1008.76] | [1340.81-1501.37] |  |
| **Hospital Service Cost** | 336.83 | 291.43 | 431.00 | <0.001 |
|  | [317.19-356.48] | [267.31-315.55] | [401.46-460.55] |  |
| Mean [95% CI]^c^ | | | | |
| **Total Cost** | 5689.87 | 4882.07 | 7365.49 | <0.001 |
|  | [5397.65-5982.10] | [4537.44-5226.69] | [6908.89-7822.09] |  |
| **Material Cost** | 1365.37 | 1096.52 | 1923.05 | <0.001 |
|  | [1253.19-1477.55] | [989.48-1203.56] | [1677.27-2168.83] |  |
| **Medication Cost** | 1472.72 | 1284.04 | 1864.09 | <0.001 |
|  | [1380.25-1565.19] | [1172.68-1395.41] | [1712.76-2015.43] |  |
| **Surgery Cost** | 763.95 | 709.91 | 876.06 | <0.001 |
|  | [737.25-790.65] | [676.61-743.20] | [836.17-915.96] |  |
| **Treatment Cost** | 459.62 | 389.79 | 604.48 | <0.001 |
|  | [425.48-493.77] | [347.03-432.56] | [554.25-654.71] |  |
| **Examination Cost** | 1236.93 | 1063.02 | 1597.67 | <0.001 |
|  | [1177.00-1296.85] | [992.23-1133.80] | [1506.59-1688.74] |  |
| **Hospital Service Cost** | 377.55 | 326.52 | 483.39 | <0.001 |
|  | [355.27-399.82] | [298.96-354.08] | [450.52-516.25] |  |
| **Note:** Values are expressed as mean [95% CI].  ^a^ Comparisons are made between the rectum group and the colon group using the Jonckheere-Terpstra Test. Results are presented as P-values.  ^b^ Costs are measured by EUR (€) according to the the annual average exchange rate of each given year.  ^c^ Costs are measured by USD($) according to the the annual average exchange rate of each given year.. | | | | |

**Supplementary Table 2.** Mean Cost of Rectal Cancer and Colon Cancer by Cost Category and Demographic Variables Measured in EUR

|  | **Total Cost** | **Material Cost** | **Medication Cost** | **Surgery Cost** | **Treatment Cost** | **Examination Cost** | **Hospital Service Cost** |
| --- | --- | --- | --- | --- | --- | --- | --- |
| **Rectal Cancer** | | | | | | | |
| **Total** | 4354.65 | 982.02 | 1141.1 | 635.18 | 347.6 | 946.55 | 291.43 |
| **Gender** |  |  |  |  |  |  |  |
| Male | 4575.80 | 1054.68 | 1212.81 | 640.62 | 366.04 | 986.45 | 302.63 |
| Female | 3962.98 | 853.34 | 1014.11 | 625.55 | 314.96 | 875.89 | 271.60 |
| P-value | 0.015 | 0.024 | 0.004 | 0.647 | 0.068 | 0.050 | 0.017 |
| **Age Group** |  |  |  |  |  |  |  |
| ≤59 | 3470.93 | 718.63 | 987.62 | 550.68 | 248.64 | 711.0 | 239.12 |
| 60-79 | 3921.66 | 835.47 | 1059.36 | 582.32 | 318.52 | 847.98 | 269.49 |
| ≥80 | 6266.04 | 1593.23 | 1488.60 | 845.16 | 515.60 | 1415.96 | 395.81 |
| P-value | <0.001 | <0.001 | <0.001 | <0.001 | <0.001 | <0.001 | <0.001 |
| **TCM** |  |  |  |  |  |  |  |
| Yes | 5611.15 | 1296.64 | 1446.21 | 713.44 | 508.69 | 1250.37 | 376.96 |
| No | 4158.57 | 932.92 | 1093.49 | 622.97 | 322.47 | 899.14 | 278.08 |
| P-value | 0.001 | 0.005 | 0.012 | 0.012 | 0.003 | <0.001 | <0.001 |
| **Cancer Stage** |  |  |  |  |  |  |  |
| TNM I | 3412.44 | 724.03 | 882.79 | 543.86 | 255.83 | 757.04 | 234.01 |
| TNM II | 4141.60 | 895.70 | 1097.31 | 619.44 | 329.99 | 890.91 | 297.46 |
| TNM III | 4571.54 | 1066.20 | 1147.86 | 682.17 | 358.12 | 1019.73 | 288.17 |
| TNM IV | 6720.04 | 1619.82 | 1949.06 | 748.46 | 609.54 | 1347.08 | 440.86 |
| P-value | <0.001 | <0.001 | <0.001 | <0.001 | <0.001 | <0.001 | <0.001 |
| **Colon Cancer** | | | | | | | |
| **Total** | 6565.31 | 1720.83 | 1656.6 | 781.81 | 539.48 | 1421.09 | 431 |
| **Gender** |  |  |  |  |  |  |  |
| Male | 6512.89 | 1737.43 | 1659.56 | 788.33 | 515.07 | 1390.15 | 408.49 |
| Female | 6658.49 | 1691.32 | 1651.34 | 770.22 | 582.87 | 1476.09 | 471.03 |
| P-value | 0.889 | 0.376 | 0.320 | 0.610 | 0.286 | 0.391 | 0.363 |
| **Age Group** |  |  |  |  |  |  |  |
| ≤59 | 6536.37 | 2428.97 | 1396.80 | 747.55 | 430.48 | 1151.00 | 374.11 |
| 60-79 | 6769.07 | 1780.05 | 1778.86 | 820.69 | 540.51 | 1389.24 | 447.85 |
| ≥80 | 6280.98 | 1425.46 | 1557.98 | 736.11 | 570.34 | 1547.06 | 423.68 |
| P-value | 0.516 | 0.506 | 0.532 | 0.082 | 0.001 | 0.002 | 0.272 |
| **TCM** |  |  |  |  |  |  |  |
| Yes | 7468.41 | 2053.99 | 1819.54 | 828.89 | 607.02 | 1655.00 | 486.66 |
| No | 6306.33 | 1625.29 | 1609.88 | 768.31 | 520.11 | 1354.01 | 415.04 |
| P-value | 0.003 | 0.066 | 0.025 | 0.042 | 0.047 | <0.001 | 0.020 |
| **Cancer Stage** |  |  |  |  |  |  |  |
| TNM I | 6217.13 | 1676.20 | 1432.39 | 854.82 | 469.45 | 1317.50 | 432.96 |
| TNM II | 6404.82 | 1566.96 | 1672.76 | 760.95 | 539.57 | 1424.39 | 430.75 |
| TNM III | 6958.63 | 2062.65 | 1683.73 | 784.80 | 547.22 | 1442.69 | 426.86 |
| TNM IV | 6388.92 | 1404.12 | 1735.28 | 767.12 | 576.62 | 1448.33 | 438.51 |
| P-value | 0.477 | 0.096 | 0.787 | 0.447 | 0.559 | 0.912 | 0.454 |
| **Note:** Costs are measured by EUR (€) according to the the annual average exchange rate of each given year.  Comparisons between groups are conducted using the Jonckheere-Terpstra Test. Results are presented as P-values. | | | | | | | |

**Supplementary Table 3.** Mean Cost of Rectal Cancer and Colon Cancer by Cost Category and Demographic Variables Measured in USD

|  | **Total Cost** | **Material Cost** | **Medication Cost** | **Surgery Cost** | **Treatment Cost** | **Examination Cost** | **Hospital Service Cost** |
| --- | --- | --- | --- | --- | --- | --- | --- |
| **Rectal Cancer** | | | | | | | |
| **Total** | 4882.07 | 1096.52 | 1284.04 | 709.91 | 389.79 | 1063.02 | 326.52 |
| **Gender** |  |  |  |  |  |  |  |
| Male | 5118.55 | 1173.41 | 1363.17 | 714.17 | 409.57 | 1105.38 | 338.51 |
| Female | 4463.26 | 960.36 | 1143.91 | 702.36 | 354.76 | 987.99 | 305.28 |
| P-value | 0.019 | 0.030 | 0.006 | 0.702 | 0.076 | 0.059 | 0.022 |
| **Age Group** |  |  |  |  |  |  |  |
| ≤59 | 3874.83 | 797.09 | 1105.46 | 613.49 | 78.83 | 796.82 | 266.59 |
| 60-79 | 4385.75 | 928.55 | 1191.29 | 649.01 | 356.27 | 949.19 | 301.58 |
| ≥80 | 7067.31 | 1794.64 | 1682.78 | 950.85 | 580.30 | 1599.38 | 445.64 |
| P-value | <0.001 | <0.001 | <0.001 | <0.001 | <0.001 | <0.001 | <0.001 |
| **TCM** |  |  |  |  |  |  |  |
| Yes | 6285.85 | 1460.63 | 1617.58 | 797.57 | 566.14 | 1402.69 | 419.84 |
| No | 4663.01 | 1039.70 | 1617.57 | 696.23 | 362.27 | 1010.01 | 311.96 |
| P-value | 0.001 | 0.008 | 0.014 | 0.025 | 0.003 | <0.001 | <0.001 |
| **Cancer Stage** |  |  |  |  |  |  |  |
| TNM I | 3843.55 | 816.99 | 995.04 | 611.53 | 287.60 | 854.02 | 262.01 |
| TNM II | 4625.11 | 994.84 | 1229.97 | 688.78 | 368.54 | 997.49 | 332.81 |
| TNM III | 5119.26 | 1187.47 | 1291.88 | 761.91 | 400.94 | 1144.07 | 322.31 |
| TNM IV | 7564.85 | 1813.83 | 2202.63 | 839.44 | 688.66 | 1516.18 | 497.99 |
| P-value | <0.001 | <0.001 | <0.001 | <0.001 | <0.001 | <0.001 | <0.001 |
| **Colon Cancer** | | | | | | | |
| **Total** | 7365.49 | 1923.05 | 1864.09 | 876.06 | 604.48 | 1597.67 | 483.39 |
| **Gender** |  |  |  |  |  |  |  |
| Male | 7335.05 | 1952.87 | 1873.26 | 886.46 | 579.67 | 1566.87 | 460.15 |
| Female | 7419.60 | 1870.03 | 1847.81 | 857.58 | 648.59 | 1652.43 | 524.70 |
| P-value | 0.765 | 0.348 | 0.709 | 0.727 | 0.315 | 0.463 | 0.375 |
| **Age Group** |  |  |  |  |  |  |  |
| ≤59 | 7208.01 | 2661.85 | 1558.26 | 818.84 | 476.05 | 1270.07 | 414.11 |
| 60-79 | 7577.81 | 1984.89 | 1997.01 | 917.78 | 604.49 | 1558.15 | 501.47 |
| ≥80 | 7107.04 | 1614.83 | 1763.83 | 833.09 | 642.59 | 1751.73 | 477.96 |
| P-value | 0.360 | 0.645 | 0.386 | 0.095 | <0.001 | <0.001 | 0.141 |
| **TCM** |  |  |  |  |  |  |  |
| Yes | 8384.29 | 2282.89 | 2057.14 | 932.10 | 679.01 | 1865.88 | 546.81 |
| No | 7073.34 | 1819.86 | 1808.74 | 860.00 | 583.11 | 1520.76 | 465.20 |
| P-value | 0.002 | 0.062 | 0.022 | 0.056 | 0.048 | <0.001 | 0.014 |
| **Cancer Stage** |  |  |  |  |  |  |  |
| TNM I | 6948.78 | 1861.75 | 1610.76 | 950.15 | 525.85 | 1476.96 | 486.40 |
| TNM II | 7171.50 | 1748.43 | 1880.90 | 850.05 | 603.20 | 1596.25 | 481.51 |
| TNM III | 7832.21 | 2313.27 | 1900.74 | 881.41 | 616.24 | 1627.77 | 480.16 |
| TNM IV | 7166.90 | 1567.05 | 1944.05 | 867.28 | 643.08 | 1631.19 | 491.85 |
| P-value | 0.586 | 0.115 | 0.634 | 0.504 | 0.712 | 0.833 | 0.547 |
| **Note:** Costs are measured by USD ($) according the the annual average exchange rate of each given year.  Comparisons between groups are conducted using the Jonckheere-Terpstra Test. Results are presented as P-values. | | | | | | | |
